# Nuclear intron sequence analysis and its implications for molecular systematics of Asian zoonotic blood flukes (*Schistosoma* spp.) (Trematoda: Schistosomatidae)

**DOI:** 10.1016/j.crpvbd.2025.100338

**Published:** 2025-11-22

**Authors:** Chairat Tantrawatpan, Wanchai Maleewong, Tongjit Thanchomnang, Warayutt Pilap, Naruemon Bunchom, Chavanut Jaroenchaiwattanachote, Ross H. Andrews, Takeshi Agatsuma, Weerachai Saijuntha

**Affiliations:** a Division of Cell Biology, Department of Preclinical Sciences, Faculty of Medicine, and Center of Excellence in Stem Cell Research and Innovation, Thammasat University, Rangsit Campus, Pathum Thani, 12120, Thailand; b Department of Parasitology, Faculty of Medicine, and Mekong Health Science Research Institute, Khon Kaen University, Khon Kaen, 40002, Thailand; c Biomedical Science Research Unit, Faculty of Medicine, Mahasarakham University, Maha Sarakham, 44000, Thailand; d Walai Rukhavej Botanical Research Institute, Mahasarakham University, Maha Sarakham, 44150, Thailand; e Center of Excellence in Biodiversity Research, Mahasarakham University, Maha Sarakham, 44150, Thailand; f Department of Tropical Medicine and Malaria, National Institute of Global Health and Medicine, Japan Institute for Health Security, Tokyo, Japan; g SATREPS Project for Parasitic Diseases (JICA/AMED), Vientiane, Laos; h Department of Surgery & Cancer, Faculty of Medicine, Imperial College, London, United Kingdom; i Department of Environmental Medicine, Kochi Medical School, Kochi University, Oko, Nankoku, Kochi, 783-8505, Japan

**Keywords:** Zoonoses, Schistosomiasis, Genetic markers, Taurocyamine kinase, Genetic variation, Genetic differentiation

## Abstract

This study explored genetic variation among and within *Schistosoma japonicum*, *S. mekongi*, and *S. malayensis* populations using two intronic regions of the taurocyamine kinase (TK) gene, the bridge intron (TkBridInt) and intron 6 of domain 1 (TkD1Int6). Both introns were successfully amplified across all species and confirmed as true intronic sequences. Sequence analysis of *S. japonicum* revealed high nucleotide polymorphism in both introns, with the population from Sorsogon (Philippines) exhibiting the highest genetic diversity. Haplotype network analysis indicated several region-specific haplotypes. Pairwise *F*_ST_ estimates demonstrated significant genetic differentiation among geographical populations, reflecting limited gene flow. The observed low intraspecific but clear interspecific genetic distances further support distinct population structure. Notably, *S. malayensis* was genetically closer to *S. mekongi* than to *S. japonicum*. These findings highlight the potential of intron sequences in detecting intra- and interspecific variation. The findings underscore the importance of applying these markers to more recently collected and geographically comprehensive samples to better elucidate host-associated genetic structuring and transmission dynamics of these zoonotic schistosomes.

## Highlights

• Two introns of the taurocyamine kinase gene were characterized in *Schistosoma japonicum*, *S. mekongi*, and *S. malayensis.*
• Both introns (TkBridInt and TkD1Int6) effectively distinguished these three species.
• Intraspecific variation was observed in *S. japonicum.*
• Preliminary geographical structuring was observed among *S. japonicum* populations.
• These intron markers offer valuable tools for studying the molecular systematics of zoonotic schistosomes.

## Graphical abstract

**Figure:**
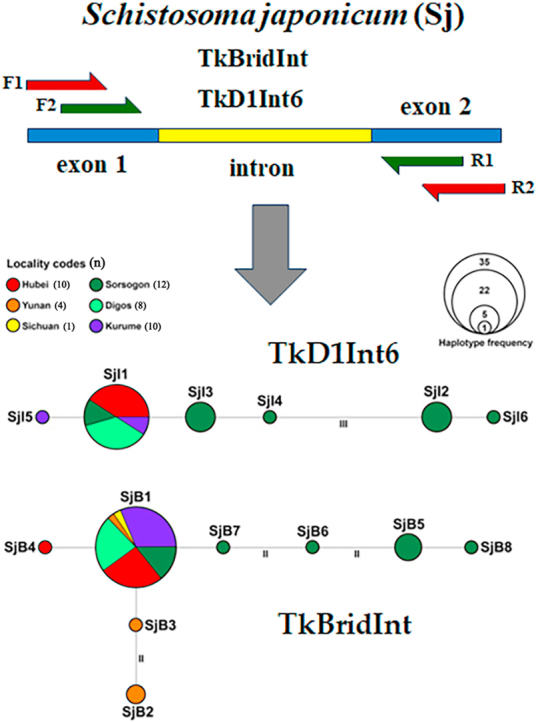

## 1 Introduction

Parasitic infections pose a substantial global health burden, particularly in developing countries, where they significantly contribute to morbidity and mortality, often exceeding other infectious diseases. Among these, helminths (parasitic worms) represent a critical group responsible for a wide spectrum of human and animal diseases. While over 287 helminth species have been reported to infect humans, approximately 95% are classified as zoonotic, meaning they can be transmitted from animals to humans (Bruschi, 2014). Approximately 100 zoonotic helminth species are known to cause either no symptoms or only mild illness in humans, with only a minority responsible for severe or life-threatening diseases (Macpherson and Craig, 1991). Of these, blood flukes (*Schistosoma* spp.) transmitted through contact with freshwater sources contaminated with their infective cercarial stage are of paramount global health concern. Globally, schistosomiasis remains a major public health concern, affecting more than 250 million people and placing over 800 million at risk of infection, with the highest burden occurring in sub-Saharan Africa and parts of Asia (WHO, 2022; Buonfrate et al., 2025).

In Asia, three major *Schistosoma* species with confirmed human pathogenicity, namely *Schistosoma japonicum*, *S. mekongi*, and *S. malayensis*, are of significant public health concern (Gordon et al., 2019). *Schistosoma japonicum* outbreaks have been reported infecting humans and animals across East Asia, including China, Japan, the Philippines, and Indonesia, with Myanmar being an emerging focus. *Schistosoma mekongi* is endemic to the Mekong River basin (Lao PDR, Cambodia, and Thailand), where it infects both humans and animals. *Schistosoma malayensis*, found in Malaysia, primarily infects rodents, though occasional human infections occur (Gordon et al., 2019). These species exhibit complex life cycles involving specific snail intermediate hosts and multiple mammalian definitive hosts, posing considerable challenges for disease control and elimination. Among these three species, *S. japonicum* is the most widespread and genetically diverse, while *S. mekongi* and *S. malayensis* are geographically restricted but equally zoonotic (Gordon et al., 2019).

Advances in molecular systematic tools have profoundly enhanced our understanding of the reproduction, genetic diversity, and evolution of medically important parasitic worms (Olson and Tkach, 2005). Genetic markers are commonly used in molecular research, and their effectiveness is determined by the extent of sequence variation, which influences their applicability. As a result, selecting an appropriate genetic marker for a particular purpose can be challenging due to differences in their characteristics and how they are utilized in various studies (Chan et al., 2021). Despite this progress, ongoing research continues to focus on developing robust molecular markers for the identification and classification of *S. japonicum* and other Asian schistosomes, including *S. mekongi* and *S. malayensis*.

Historically, mitochondrial and nuclear coding genes, microsatellite markers have been widely used to explore genetic diversity, population genetics, molecular systematics, and host specificity in *Schistosoma* spp. (e.g. Le et al., 2002; Ray et al., 2021). These markers have provided valuable insights into transmission dynamics and host-parasite associations. However, intron regions of nuclear genes, such as those from the taurocyamine kinase (TK) gene, evolve more rapidly than coding regions and often display higher polymorphism, providing greater resolution for fine-scale population genetic and phylogenetic analyses. Their widespread presence across diverse parasitic taxa further makes them effective markers for studying genetic diversity and differentiation in parasitic trematodes (Saijuntha et al., 2016, 2018, 2020; Tantrawatpan et al., 2023).

The TK gene plays an essential role in the survival of flatworms by producing enzymes necessary for ATP/ADP hydrolysis in the energy synthesis process (Ellington, 2001). Complete nucleotide sequences of the TK gene have been obtained from several trematode species, including *Clonorchis sinensis* (Xiao et al., 2013), *Paragonimus westermani* (Jarilla et al., 2009), and *S. japonicum* (Tokuhiro et al., 2013). The TK gene structure in flatworms typically comprises two conserved domains separated by a bridge intron. Each domain contains two to three introns, which are known to be highly variable and exhibit considerable heterozygosity (Jarilla et al., 2013). Previous studies have successfully demonstrated the utility of these intron regions for investigating genetic variation and population structure in various trematode species, including *Paragonimus* spp. (Saijuntha et al., 2016), *Fasciola* spp. (Saijuntha et al., 2018), *Echinostoma* spp. (Saijuntha et al., 2020), as well as *Opisthorchis viverrini* and *C. sinensis* (Tantrawatpan et al., 2023). Thus, this study aimed to characterize intron regions of the TK gene to elucidate species-level distinctions and assess the potential use of these markers for examining intraspecific genetic variation and conducting population genetic analyses among medically important Asian *Schistosoma* species, specifically *S. japonicum*, *S. mekongi*, and *S. malayensis*.

## 2 Materials and methods

### 2.1 DNA samples and primer designs

All DNA samples of blood flukes used in this study were kindly provided by Prof. Takeshi Agatsuma (Kochi Medical School, Japan) and were originally collected between 1997 and 2000. The quantity and quality of DNA extracted from several *S. japonicum* samples were suboptimal, with low concentrations, limited volumes, and evidence of degradation, likely due to prolonged storage. A total of 46 DNA samples of *S. japonicum* originating from various localities in Japan*,* China, and the Philippines were included, along with two samples of *S. mekongi* from Lao PDR*,* and two samples of *S. malayensis* from Malaysia (Table 1). To amplify two intron regions of the TK gene, specifically intron 6 of domain 1 (TkD1Int6) and the bridge intron (TkBridInt), we designed primer pairs targeting conserved exon-flanking regions based on the full-length TK gene sequence of *S. japonicum* (GenBank: AB778506) (Table 2). In cases where amplification with the first primer pair (F1 and R1) resulted in weak or failed PCR products, a second primer pair (F2 and R2) (Table 2) was used to amplify both intron regions by a subsequent PCR reaction.

**Table 1:** Details of sampling localities and the number of samples of Asian *Schistosoma* spp. examined in this study.

Species	Locality	Country	Number of samples			PCR^a^	
			Female	Male	Total	TkD1Int6	TkBridInt
*S. japonicum*	Kurume	Japan	5	5	10	3	10
	Sorsogon	Philippines	7	5	12	12	12
	Digos	Philippines	4	4	8	8	8
	Hubei	China	0	10	10	9	10
	Yunnan	China	0	4	4	–	4
	Sichuan	China	1	0	1	–	1
*S. malayensis*	Baling	Malaysia	0	2	2	2	2
*S. mekongi*	Khong Island	Lao PDR	0	2	2	2	2

*Note*: −, not analyzed.

a Number of successfully amplified samples for each intron.

**Table 2:** List of primers used for PCR amplification of TkD1Int6 and TkBridInt regions.

Intron region	Intron name	Primer name	Primer sequence (5′-3′)
Domain 1 intron 6	TkD1Int6	SjTkD1Int6F1	ACTGAAATTTGCATTCAGTGAT
		SjTkD1Int6R1	TCAGTAAGACCAAGACGGCGTT
		SjTkD1Int6F2	ATTCAGTGATCGTTTTGGGTTT
		SjTkD1Int6R2	CCAAGACGGCGTTTATTACTTAA
Bridge intron	TkBridInt	SjBridgeIntF1	GGTATTTACGATTTAAGTAATA
		SjBridgeIntR1	ATTTCTTAATTATTTCTGGAGT
		SjBridgeIntF2	TTAAGTAATAAACGCCGTCTTGG
		SjBridgeIntR2	ATTTCTTAATTATTTCTGGAGT

### 2.2 PCR analysis and DNA sequencing

To amplify each intron region, gradient PCR was used to optimize suitable conditions using an annealing temperature within the range of 45–55 °C. An annealing temperature of 50 °C was identified as optimal for both introns. The PCR condition for both intron regions consisted of an initial denaturation at 94 °C for 3 min, followed by 35 cycles of denaturation at 94 °C for 30 s, annealing at 50 °C for 30 s, and extension at 72 °C for 1 min, with a final extension step at 72 °C for 5 min. In the case of low concentration (no/faint band), 1 μl of the first PCR product was used as the DNA template for the second PCR using the same conditions as used with the first PCR. The PCR mixture contained 1× TaKaRa Ex PCR buffer, 0.2 mM dNTPs (each), 0.2 μM of each primer, and 1.0 U of TaKaRa Ex Taq polymerase (Takara Bio Inc., Shiga, Japan). Subsequently, the PCR product underwent electrophoresis in 1% agarose gel and was visualized with GelRed^TM^ Nucleic Acid Gel Stain (Biotium, Inc., Hayward, CA). PCR product (∼1000–1200 bp) was cut for gel purification using E.Z.N.A.® Gel Extraction kit (Omega bio-tek, Norcross, GA, USA), and was subsequently used for DNA sequencing. The purified PCR products were sequenced using the Sanger sequencing platform *via* a commercial service provider (ATGC Co., Ltd., Pathum Thani, Thailand). The newly generated sequences were deposited in the GenBank database under the accession numbers PX527122-PX527160 (TkD1Int6) and PX527071-PX527120 (TkBridInt).

### 2.3 Data analysis

Both intron regions were sequenced bidirectionally, and chromatograms were carefully inspected and edited using BioEdit version 7.2.6 (Hall, 1999). The exon regions were identified by BLAST searches in NCBI to confirm that the amplified fragments corresponded to the true intronic region of the TK gene. Thereafter, those exon regions were trimmed out before being used in intron sequence analysis. Heterozygous nucleotide positions were identified based on the presence of clear double peaks in both forward and reverse sequences. These positions were treated as distinct haplotypes, with each haplotype defined by the specific nucleotide variants at the heterozygous sites. The intron sequences were aligned using the ClustalW program (Larkin et al., 2007) to compare and search for the variable sites, including microsatellite and insertion/deletion (indel) regions. Diversity indices and haplotype data were calculated and generated using the DnaSp v5 program (Librado and Rozas, 2009). Haplotype networks were constructed using TkBridInt and TkD1Int6 sequences of *S. japonicum* from different geographical localities in the Network program version 10.2 (https://www.fluxus-engineering.com/) based on a median-joining method (Bandelt et al., 1999) using all sequences generated in this study. Genetic differences between *S. japonicum*, *S. malayensis*, and *S. mekongi*, as well as among populations within *S. japonicum*, were calculated based on the uncorrected p-distance and Kimura-2-parameter (K2P) (Kimura, 1980) using the program MEGA 11 (Kumar et al., 2018). Genetic differentiation (*F*_ST_) analysis was conducted using the Arlequin program version 3.5.2.2 (Excoffier and Lischer, 2010).

## 3 Results

### 3.1 Intron sequence variation

**Fig. 1:**
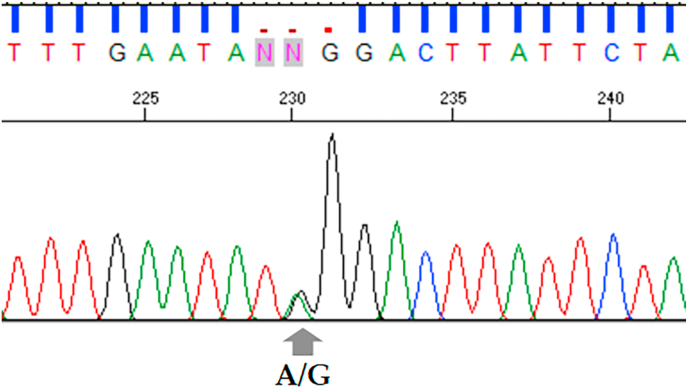
Heterozygous pattern observed in a *Schistosoma japonicum* sample from Sorsogon, showing double peaks (A/G) at a specific nucleotide site of the TkD1Int6 sequence chromatogram obtained from Sanger sequencing.

The TkBridInt and TkD1Int6 regions were successfully amplified and characterized in all *Schistosoma* species examined, *S. japonicum*, *S. mekongi*, and *S. malayensis*, and confirmed to be true intronic regions of the TK gene by the existence of its exon regions on both sides. However, TkD1Int6 sequences could not be obtained for the *S. japonicum* specimens from Sichuan and Yunnan because the available DNA was of limited quantity and was further depleted during early PCR optimization steps. In total, 46 sequences of *S. japonicum* were analyzed for the TkBridInt region and 35 sequences for the TkD1Int6 region. Nine variable nucleotide positions (1.06%) were detected within the 852-bp length of the TkBridInt region (Supplementary Table S1). Among these, four purine transitions, two pyrimidine transitions, and three transversions were observed. On the other hand, five nucleotide variable positions (0.51%) within 973 bp of TkD1Int6 were observed. These variable positions represented one pyrimidine transition (133), two purine transitions, and two transversions. Additionally, at position 445 of TkD1Int6 sequences, three variants of nucleotide (purine transition and transversion) were found (Supplementary Table S1). Within the TkD1Int6 region of *S. japonicum*, a tri-nucleotide microsatellite insertion (TAA) was detected, suggestive of heterozygosity in several samples from Digos. The heterozygous samples of TkD1Int6 can be used for further analysis by excluding this repeated sequence. Additionally, a heterozygous site combined between base A and G (Fig. 1) of TkD1Int6 was specifically observed in several samples from Sorsogon.

### 3.2 Genetic variation of *Schistosoma japonicum*

The genetic diversity indices based on the intron regions TkBridInt and TkD1Int6 varied among *S. japonicum* populations from different geographical localities in China, Japan, and the Philippines (Table 3). For the TkBridInt region, the number of haplotypes ranged from one to five, with haplotype diversity (*Hd*) values ranging from 0 to 0.833. The greatest nucleotide diversity (*π* = 0.0033 ± 0.0004) was observed in the Sorsogon population, which also exhibited the largest number of polymorphic sites (*S* = 6) and unique haplotypes (*Uh* = 4). In contrast, no variation was detected in the Digos and Kurume populations (*Hd* = 0), indicating a lack of intron diversity at this locus. For the TkD1Int6 region, five haplotypes were identified across 35 sequences, with haplotype diversity values ranging from 0 in Digos and Hubei to 0.781 in the Sorsogon population. The Sorsogon population again showed the greatest genetic diversity, with five polymorphic sites, five haplotypes (four of which were unique), and the greatest nucleotide diversity (*π* = 0.0026 ± 0.0003). A moderate level of genetic variation was observed in the Kurume population (*Hd* = 0.667), while no variation was detected in Hubei and Digos. Overall, total haplotype and nucleotide diversity were comparable between the two introns, with *Hd* = 0.418 and *π* = 0.0017 for TkBridInt, and *Hd* = 0.578 and *π* = 0.0017 for TkD1Int6, respectively (Table 3).

**Table 3:** Diversity indices of the sequences of TkBridInt and TkD1Int6 in *Schistosoma japonicum* populations from various localities.

Populations	TkBridInt						TkD1Int6					
	*n*	*S*	*H*	*Uh*	*Hd* ± SD	*π* ± SD	*n*	*S*	*H*	*Uh*	*Hd* ± SD	*π* ± SD
Kurume	11	0	1	0	0.000 ± 0.000	0.0000 ± 0.0000	3	1	2	1	0.667 ± 0.314	0.0007 ± 0.6667
Sichuan	1	0	1	0	0.000 ± 0.000	0.0000 ± 0.0000	–	–	–	–	–	–
Yunnan	4	3	3	2	0.833 ± 0.222	0.0022 ± 0.0006	–	–	–	–	–	–
Hubei	10	1	2	1	0.200 ± 0.154	0.0002 ± 0.0002	9	0	1	0	0.000 ± 0.000	0.0000 ± 0.0000
Digos	8	0	1	0	0.000 ± 0.001	0.0000 ± 0.0001	8	0	1	0	0.000 ± 0.000	0.0000 ± 0.0000
Sorsogon	12	6	5	4	0.758 ± 0.093	0.0033 ± 0.0004	15	5	5	4	0.781 ± 0.064	0.0026 ± 0.0003
Total	46	9	8	7	0.418 ± 0.090	0.0017 ± 0.0005	35	5	6	5	0.578 ± 0.085	0.0017 ± 0.0004

*Abbreviations*: *n*, number of DNA samples; *S*, number of segregations sites; *H*, number of haplotypes; *Uh*, number of unique haplotypes; *Hd*, haplotype diversity; *π*, nucleotide diversity; SD, standard deviation; –, not analyzed.

### 3.3 Haplotype network

**Fig. 2:**
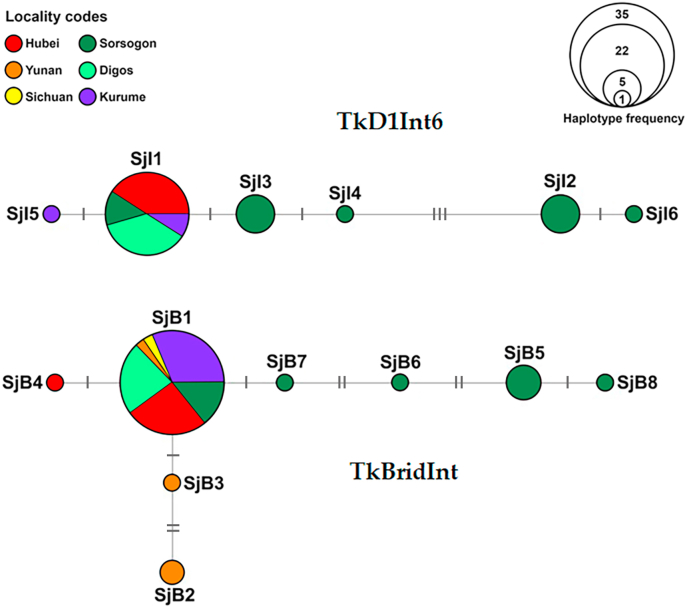
Haplotype networks of *Schistosoma japonicum* sequences based on TkD1Int6 (SjI1-SjI6) and TkBridInt (SjB1-SjB8) sequences from various localities. The size of each circle is proportional to the number of samples sharing that haplotype. The number of mutational steps between haplotypes is indicated by hatch marks along the connecting branches.

Haplotype networks based on the TkD1Int6 and TkBridInt intron regions revealed distinct haplotype compositions among *S. japonicum* populations from different localities (Fig. 2). For the TkD1Int6 region, six haplotypes (SjI1-SjI6) were identified. Haplotype SjI1 was observed in individuals from Hubei, Sorsogon, Digos, and Kurume. Haplotypes SjI2, SjI3, and SjI4 occurred primarily in Sorsogon, while haplotypes SjI5 and SjI6 were specifically detected in Kurume and Sorsogon, respectively. The haplotypes were separated by 1–3 mutational steps. For the TkBridInt region, eight haplotypes (SjB1-SjB8) were detected. Haplotype SjB1 occurred in individuals from Hubei, Yunnan, Sichuan, Sorsogon, Digos, and Kurume. Haplotypes SjB2 and SjB3 were found only in Yunnan, haplotype SjB4 was found only in Hubei, and haplotypes SjB5-SjB8 were restricted to Sorsogon. Most haplotypes were separated by one or two mutational steps (Fig. 2).

### 3.4 Genetic differentiation

Pairwise genetic differentiation (*F*_ST_), assessed using the TkD1Int6 and TkBridInt intron sequences, revealed significant population structure within *S. japonicum* across several geographical localities (Table 4). Both markers indicated strong and statistically significant differentiation between Hubei and Sorsogon (*P*-value < 0.001). Additionally, the TkBridInt marker revealed significant differentiation between Digos and Sorsogon. The TkD1Int6 analysis showed that Yunnan was significantly differentiated from Digos, Hubei, and Sorsogon, while Kurume was significantly differentiated from both Yunnan and Sorsogon (Table 4). Pairwise genetic distances, calculated using both uncorrected p-distance and K2P models (Table 5), demonstrated distinct patterns of divergence within and among *S. japonicum*, *S. malayensis*, and *S. mekongi*. Intraspecific divergence within *S. japonicum* was extremely low, with genetic distances consistently ranging from 0 to 0.0045 for both models. In contrast, higher interspecific distances were observed when comparing *S. japonicum* with its congeners. Specifically, the genetic distances between *S. japonicum* and *S. malayensis* ranged from 0.1536 to 0.1571 based on p-distance and from 0.1367 to 0.1395 based on K2P, while comparisons with *S. mekongi* yielded values of 0.1542–0.1577 for p-distance and 0.1373 to 0.1401 for K2P (Table 5).

**Table 4:** Genetic differentiation (*F*_ST_) among different populations of *Schistosoma japonicum*, based on TkBridInt (lower triangle) and TkD1Int6 (upper triangle) sequences.

Locality	Kurume	Sichuan	Yunnan	Digos	Hubei	Sorsogon
Kurume	–	n/a	n/a	0.2456	0.3793	0.2456
Sichuan	0.0000	–	n/a	n/a	n/a	n/a
Yunnan	0.7007∗	0.0476	–	n/a	n/a	n/a
Digos	0.0000	0.0000	0.6356∗	–	0.0000	0.3928∗∗∗
Hubei	0.0100	1.0000	0.6136∗∗	0.0242	–	0.4080∗
Sorsogon	0.4164∗	0.1394	0.3476∗∗	0.3679∗	0.3885∗∗∗	–

*Note*: ∗*P* < 0.05; ∗∗*P* < 0.01; ∗∗∗*P* < 0.001.

*Abbreviation*: n/a, not available.

**Table 5:** Genetic differences based on the TkD1Int6 sequence, calculated using the uncorrected p-distance (below the diagonal) and K2P (above the diagonal), for comparisons among different populations of *S. japonicum* as well as with *S. malayensis* and *S. mekongi*.

	*S. japonicum*						*S. malayensis*	*S. mekongi*
	Kurume	Sichuan	Yunnan	Digos	Hubei	Sorsogon		
Kurume	–	0.0000	0.0021	0.0000	0.0001	0.0029	0.1370	0.1376
Sichuan	0.0000	–	0.0021	0.0000	0.0001	0.0029	0.1370	0.1376
Yunnan	0.0021	0.0021	–	0.0021	0.0022	0.0045	0.1367	0.1373
Digos	0.0000	0.0000	0.0021	–	0.0001	0.0029	0.1370	0.1376
Hubei	0.0001	0.0001	0.0022	0.0001	–	0.0031	0.1368	0.1375
Sorsogon	0.0029	0.0029	0.0045	0.0029	0.0031	–	0.1395	0.1401
*S. malayensis*	0.1540	0.1540	0.1536	0.1540	0.1538	0.1571	–	0.0187
*S. mekongi*	0.1546	0.1546	0.1542	0.1546	0.1544	0.1577	0.0190	–

These values support species-level divergence among *S. japonicum*, *S. malayensis*, and *S. mekongi*. Notably, the genetic distance between *S. malayensis* and *S. mekongi* was substantially lower, ranging from 0.0187 to 0.0190 under both models (Table 5), indicating a closer evolutionary relationship between these two species than with *S. japonicum.*

## 4 Discussion

Some *S. japonicum* DNA samples, particularly from Yunnan and Sichuan, were of low quality or depleted during PCR optimization, limiting amplification of TkD1Int6. Consequently, the restricted sample size and geographical coverage may affect inferences on molecular systematics and population structure. Additionally, our DNA samples were collected more than 25 years ago, which may not fully reflect the current patterns of genetic variation and population structure of *S. japonicum*. To address these limitations, future studies should incorporate a broader and more representative sample set and apply these intron regions as informative nuclear markers to investigate genetic diversity, population structure, gene flow, and local adaptation across the full geographical range of *S. japonicum*, thereby enhancing our understanding of the evolutionary dynamics of this important zoonotic parasite.

Despite these unforeseen limitations, both intron regions characterized in this study exhibited higher levels of nucleotide polymorphism compared to commonly used nuclear DNA markers in *S. japonicum*, such as the internal transcribed spacer (ITS) region and 18S rDNA. These conventional nuclear markers are typically highly conserved and exhibit limited intraspecific variation, thereby reducing their utility for fine-scale population genetic and phylogeographic analyses (Bowles et al., 1995; Le et al., 2002). In contrast, the intron markers TkD1Int6 and TkBridInt revealed substantial sequence variability both within and among *S. japonicum* populations, highlighting their potential for resolving intraspecific genetic differentiation. When compared to mitochondrial markers such as cytochrome *c* oxidase subunit 1 (*cox*1) and NADH dehydrogenase subunit 1 (*nad*1), which are often used due to their high mutation rates and maternal inheritance (Zhao et al., 2012; Chen et al., 2015), these intron regions demonstrated comparable or even higher levels of variation in certain populations.

We found significant genetic differentiation among *S. japonicum* populations from different localities in this study. Our findings are in concordance with several previous reports that revealed strong geographical structuring and substantial genetic variation among *S. japonicum* populations across endemic regions in China and Southeast Asia. For example, studies using microsatellite markers and mitochondrial DNA sequences demonstrated clear population subdivisions between isolates from mainland China, the Philippines, and Indonesia, reflecting historical isolation, ecological differences, and host usage patterns (Shrivastava et al., 2005; Zhang et al., 2007; Yin et al., 2008). Furthermore, *S. japonicum* populations from mountainous and lake regions in China were also found to differ significantly, suggesting restricted gene flow and adaptation to local intermediate and definitive hosts (Zhou et al., 2009; Rudge et al., 2010). Previous findings, including those from our study, support the hypothesis that geographical separation and ecological specialization play crucial roles in shaping the genetic structure of *S. japonicum* populations (Yin et al., 2015; Moendeg et al., 2017).

Interestingly, we observed heterozygous patterns in the TkD1Int6 intron sequence in samples from Sorsogon, Philippines. Several samples from Digos also exhibited heterozygosity associated with a TAA repeat indel within the same intron region. While these patterns suggest the possible presence of multiple alleles within individuals, they should be interpreted cautiously, as chromatogram double peaks can have alternative explanations, such as DNA degradation or poor quality (Al-Shuhaib and Hashim, 2023). Nevertheless, these observations provide preliminary evidence of nuclear intron variation within these populations. A similar observation has been reported in *C. sinensis*, in which the intron 5 in domain 1 of TK (TkD1Int5) sequence revealed intra-individual heterozygosity, reflecting high nuclear genetic diversity and potentially ongoing genetic recombination or mixed infections in natural populations (Tantrawatpan et al., 2023). These findings imply that certain populations may harbor a higher level of genetic complexity, possibly due to co-infection by genetically distinct strains or the presence of sexual recombination in the parasite's life cycle.

The genetic variation of *S. japonicum* is shaped by the diversity, abundance, and mobility of its definitive hosts. In regions such as China and the Philippines, where multiple mammalian hosts participate in transmission, higher genetic diversity and heterozygosity have been reported, likely reflecting host-specific adaptation and differences in host migration patterns (Wang et al., 2006; Rudge et al., 2008, 2010; Long et al., 2025). Thus, host composition and sampling intensity significantly influence allele distribution and population structure in *S. japonicum* (Long et al., 2025). Comparable host-associated genetic structuring has also been observed in *O. viverrini*, where genotyping with the TkD1Int5 intron marker revealed distinct parasite genotypes potentially associated with different definitive host species, such as cats (Vaisusuk et al., 2025). Therefore, future molecular systematic and population genetic studies using the intron sequences identified here could enhance understanding of host-associated structure and transmission patterns in *S. japonicum*, as well as other closely related species.

Based on our findings, both TkD1Int6 and TkBridInt are proven to be promising genetic markers that are suitable for molecular systematic and population genetic analyses of Asian blood flukes. These intron regions were successfully amplified in all examined species and differentiated *S. japonicum*, *S. mekongi*, and *S. malayensis*. Moreover, genetic differences revealed by the TkD1Int6 analysis showed that *S. malayensis* is more closely related to *S. mekongi* than to *S. japonicum*, consistent with a previous report (Le et al., 2002). Thus, both introns appear to be reliable and informative markers for distinguishing and exploring phylogeny among the three Asian *Schistosoma* species analyzed in this study. Furthermore, these markers have the potential to be applied in future studies involving other medically important species of *Schistosoma*, such as *S. mansoni*, *S. haematobium*, and *S. intercalatum*, to assess genetic variation, elucidate phylogenetic relationships, and support species delimitation within the genus as well as genetic variation of each species.

## 5 Conclusions

This study presents the first characterization of two nuclear intron regions, TkD1Int6 and TkBridInt, from the TK gene as genetic markers for assessing genetic variation in *S. japonicum* and related species. Both introns were successfully amplified across all examined species and exhibited clear patterns of intraspecific polymorphism and interspecific divergence. Significant genetic differentiation was observed among *S. japonicum* populations from different geographical localities, aligning with previous reports of geographical structuring in this species. Compared to conventional nuclear markers, these intron regions exhibit greater levels of genetic variation, thereby supporting their utility in molecular systematics and population genetic analyses. Despite limitations due to the age and quality of available DNA samples, the findings highlight the potential of nuclear intron sequences to uncover genetic diversity, evolutionary relationships, and possible host-associated differentiation in *S. japonicum*. Future studies incorporating freshly collected specimens and expanded geographical sampling are recommended to further evaluate the broader applicability of these markers across populations and related species.

## Ethical approval

Biosafety was approved by the Institute Biosafety Committee of Thammasat University, permission no. 035/2565.

## CRediT authorship contribution statement

**Chairat Tantrawatpan:** Supervision, Conceptualization, Investigation, Methodology, Formal analysis, Writing – original draft, Writing – review & editing. **Wanchai Maleewong:** Resources, Funding acquisition, Writing – review & editing. **Tongjit Thanchomnang:** Methodology, Writing – review & editing. **Warayutt Pilap:** Investigation, Methodology. **Naruemon Bunchom:** Investigation, Methodology. **Chavanut Jaroenchaiwattanachote:** Formal analysis, Visualization. **Ross H. Andrews:** Investigation, Writing – review & editing. **Takeshi Agatsuma:** Resources, Writing – review & editing, Validation. **Weerachai Saijuntha:** Supervision, Conceptualization, Investigation, Methodology, Formal analysis, Funding acquisition, Writing – review & editing, Validation.

## Funding

This research project was financially supported by the Faculty of Medicine, Mahasarakham University. The National Research Council of Thailand (NRCT) through the High-Potential Research Team Grant Programme (grant number N42A670561), awarded to Wanchai Maleewong.

## Declaration of competing interests

The authors declare that they have no known competing financial interests or personal relationships that could have appeared to influence the work reported in this paper.

## Data Availability

All newly generated intron sequences of *S. japonicum*, *S. mekongi*, and *S. malayensis* examined in this study were deposited in GenBank under the accession numbers PX527122-PX527160 (TkD1Int6) and PX527071-PX527120 (TkBridInt).
